# Pregnancy outcomes in systemic sclerosis before and after diagnosis: a retrospective nationwide cohort study

**DOI:** 10.55730/1300-0144.6196

**Published:** 2026-03-30

**Authors:** Alper DOĞANCI, Serdar Can GÜVEN, Nuray YILMAZ ÇAKMAK, Rıfat BOZKUŞ, Salih BAŞER, Ekin DOĞANCI, Neslihan ÖZTÜRK, Şuayip BİRİNCİ, Naim ATA

**Affiliations:** 1 Division of Rheumatology, Ankara Atatürk Sanatory Education and Research Hospital, Ankara, Turkiye; 2 Division of Rheumatology, Ankara Bilkent City Hospital, Ankara, Turkiye; 3 Department of Internal Medicine, Faculty of Medicine, Ankara Yıldırım Beyazıt University, Ankara, Turkiye; 4 Department of Internal Medicine, Ankara Etlik City Hospital, Ankara, Turkiye; 5 Divison of Rheumatology, Ankara Etlik City Hospital, Ankara, Turkiye; 6 Republic of Türkiye Ministry of Health Strategy Development Department, Ankara, Turkiye; 7 Deputy Minister, Ministry of Health of the Republic of Türkiye, Ankara, Turkiye

**Keywords:** Systemic sclerosis, scleroderma, pregnancy, Robson classification, outcomes

## Abstract

**Background/aim:**

Pregnancy outcomes in women with systemic sclerosis (SSc) were evaluated in a nationwide cohort study. The impact of the disease on pregnancy outcomes was further assessed by comparing outcomes before and after the diagnosis of systemic sclerosis.

**Materials and methods:**

A retrospective, nationwide, multicenter study was conducted under the supervision of the Ministry of Health. A nationwide cohort was between 1 January 2018, and 31 October 2025, including female patients with at least two recorded ICD codes for SSc (M34 subcategories) at an interval of at least 1 month, who were identified as pregnant before (pre-SSc) or after SSc (post-SSc) based on ICD-10 codes (Z32, Z33, Z34, Z35). The initiation of data collection was set as 1 January 2018, because complete birth outcome records were available in the database from that date onward. Patients were categorized into pre-SSc and post-SSc groups according to the timing of pregnancy relative to the diagnosis of SSc.

**Results:**

Among 43,663 patients diagnosed with SSc, data from a total of 4914 pregnancies/pregnancy outcomes were retrospectively examined. Abortion was more frequent in women with SSc (11.70% in the pre-SSc group [relative risk (RR) 1.64, 95% CI 1.33–2.03], and 10.64% in the post-SSc group [RR 1.48, 95% CI 1.11–1.96]). The risk of gestational diabetes was significantly increased in both groups, with a greater increase observed in the pre-SSc group (RR 6.66); similarly, hypertensive disorders of pregnancy were more frequent in the pre-SSc group (RR 8.03). Puerperal complications were observed in 2.54% of the pre-SSc group and 3.12% (n = 22) of the post-SSc group (RR 1.85 and 2.28, respectively, compared with healthy controls).

**Conclusion:**

Our results suggest that women with SSc are at increased risk; however, successful pregnancies are possible under appropriate multidisciplinary care.

## 1. Introduction

Systemic sclerosis (SSc) is a complex, multifactorial autoimmune disease characterized by fibrosis, inflammation, and vasculopathy. The incidence in women can be up to nine times higher [1]. The average age at disease onset is in the early fifth decade; therefore, most patients with SSc have already passed reproductive age at the time of diagnosis. In recent years, the tendency to postpone pregnancy has become more widespread due to social and economic factors, increasing the likelihood of pregnancy after disease onset. Therefore, pregnancy has become an important issue that needs to be addressed in patients with SSc.

The existing literature on pregnancy in SSc reports inconsistent findings regarding pregnancy outcomes, while data on maternal morbidity remain limited. Most studies published before 1990, primarily consisting of case series, reported poor maternal and fetal outcomes, with an increased frequency of obstetric complications [2]. However, one of the two prospective studies published to date reported more favorable outcomes, with pregnancy success rates similar to those of the general population and no increase in maternal mortality [3]. However, these studies were mostly designed as survey-based investigations, and the number of patients included was limited due to the rarity of the disease [4,5]. This study includes a considerable number of patients and further evaluates outcomes both before and after the diagnosis.

In patients with systemic lupus erythematosus (SLE) and antiphospholipid syndrome (APS), obstetric complications such as recurrent miscarriage, preeclampsia, placental insufficiency and intrauterine growth restriction (IUGR) are better defined than in SSc [6,7]. SSc is a more complex disease compared with SLE and APS, as it involves the coexistence of vasculopathy, fibrosis, and autoimmunity. Therefore, disease-specific data for SSc are essential despite the availability of cumulative data from other autoimmune diseases.

A high frequency of sexual dysfunction has been reported among women with SSc, and the pathogenic mechanisms of the disease may play a major role [8]. Women with SSc should be informed in detail about the risks during pregnancy, as well as the potential social challenges associated with high-risk pregnancy. Large cohort studies are needed to provide such information.

In this study, maternal and fetal outcomes in pregnancies of women with systemic sclerosis were evaluated using a nationwide database, and outcomes before and after SSc diagnosis were compared with matched controls to further elucidate the impact of the disease on pregnancy outcomes.

## 2. Materials and methods

### 2.1. Study design

The study was designed as a retrospective, nationwide, multicenter study. The Turkish Ministry of Health National Electronic Database (e-Nabız) was used under the supervision of the Ministry of Health to extract data from the study population. The e-Nabız system was established by the Ministry of Health in 2015 as a national health information system to which only authorized individuals and institutions have access; it has a wide data capacity and covers the entire country. The e-Nabız system contains the clinical records of over 80 million individuals in Türkiye, including demographic data, recorded ICD codes, laboratory results, medication history, and comorbidities. The Ministry of Health delivers services using big data technology, and these systems are integrated, including e-Nabız and the National Healthcare Information System (NHIS) [9]. Through these integrated systems, data on all residents have been available since 1 January 2016. Furthermore, birth reports for all pregnancies ending in delivery are accessible through the national database system.

### 2.2. Study population

A nationwide cohort was created between 1 January 2018 and 31 October 2025, from female patients who had an ICD code for SSc (M34 subfractions) was entered twice at least with an interval of 1 month. In this cohort, patients with ICD codes for pregnancy (Z32, Z33, Z34, Z35) recorded before the first ICD code for SSc and those recorded after the first ICD code for SSc were classified as the pre-SSc and post-SSc pregnancy groups, respectively. The start of data collection was defined as 1 January 2018, as complete birth outcome reports were available in the database from that date onward. The cohort was evaluated retrospectively using a cross-sectional design. According to the American College of Rheumatology/European League Against Rheumatism (ACR/EULAR) systemic sclerosis classification criteria, patients were identified based on recorded findings such as anti-RNA polymerase III, anticentromere, and anti-Scl-70 positivity, as well as clinical features including Raynaud’s phenomenon, sclerodactyly, digital ulcers, telangiectasias, abnormal capillaroscopy findings, pulmonary arterial hypertension (PAH), and interstitial lung disease, as documented in epicrisis reports and outpatient clinic records. ICD codes for pregnancy-related events occurring within 9 months after the initial pregnancy-related ICD code were considered to be associated with the same pregnancy. Patients whose birth records could not be obtained and those who had pregnancies both before and after SSc diagnosis were excluded from the study.

The following characteristics of pregnancies were evaluated: comorbidities, infertility status (ICD code: N97), mode of delivery, and pregnancy- and birth-related complications, including gestational diabetes (O24), preeclampsia (O10–O16), preterm birth, miscarriage (N96.0, O26, O00.0–O07), placenta previa (O44), placental abruption (O43), bleeding during pregnancy (O20), venous complications (O22), ectopic pregnancy (O00), molar pregnancy (O08), multiple pregnancy (O30), liver disorders during pregnancy (O26.6), puerperal infections (O86), venous complications during the puerperium (O87), obstetric embolism (O88), cardiomyopathy during the puerperium (O90), cesarean section (CS) (O82), vaginal delivery (O83), forceps delivery (O81), and multiple births (O84). Pregnancies were defined as high risk if the mother was younger than 18 years or older than 35 years, or if chronic diseases or multiple pregnancy were present. Birth weight, gestational age at delivery, neonatal mortality, and Robson classification scores were also extracted from birth reports. The Robson classification system uses basic obstetric characteristics to categorize all women admitted for delivery into one of ten mutually exclusive and totally inclusive groups. Unlike other CS classification systems (e.g., those based on indications for CS), the Robson classification has gained widespread acceptance across diverse clinical settings [10]. The World Health Organization (WHO) and the International Federation of Gynecology and Obstetrics (FIGO) recommend the Robson classification as a global standard for assessing, monitoring, and comparing CS rates within and between healthcare facilities over time.

The control group consisted of pregnant women with no previously recorded ICD codes for any rheumatologic disease, including SSc, and no healthcare visits during pregnancy for complications other than routine follow-up visits to family medicine and obstetrics/gynecology outpatient clinics. The control group was individually matched on a 1:1:1 basis according to maternal age, age at delivery, and year of delivery. Treatment agents used for SSc were recorded when corresponding codes were present in the database.

### 2.3. Statistical analysis

Data were analyzed using SPSS, version 25 (IBM Corp., Armonk, NY, USA). Continuous variables were presented as either median (IQR) or mean ± SD, according to data distribution, whereas categorical variables were presented as number (%). Categorical variables were compared using the chi-square test or Fisher’s exact test, as appropriate, whereas student’s t-test was used for continuous variables. Cox regression analyses were performed to estimate the relative risk (RR) of pregnancy events compared with the control group. A p < 0.05 was considered statistically significant.

Because of the retrospective design of the study, informed consent was waived.

### 2.4. Ethical approval

This study was conducted with the permission of the Ministry of Health (approval no. 95741342-020). The study was conducted in accordance with the Declaration of Helsinki.

## 3. Results

### 3.1. Clinical characteristics

A total of 4914 women with SSc were detected female SSc patients were retrospectively analyzed. Among these a total of 2474 pregnancy outcomes from 2120 pregnant women with SSc were evaluated. A control group comprising 2440 pregnancy outcomes from 2120 pregnant women were formed from female patients without any connective tissue disease were also retrospectively analyzed. Pregnancy outcomes from women with SSc and matched controls, were compared.

Patients were categorized according to the timing of pregnancy relative to the diagnosis of SSc. A total of 1769 patients had their first pregnancy before the first ICD code for SSc and were classified as the pre-SSc group, whereas 705 patients had their first pregnancy after the first recorded ICD code for SSc and were classified as the post-SSc group.

The proportion of births with a birth weight of ≤2500 g was 11.76% in the pre-SSc group, 10.07% in the post-SSc group, and 4.88% in the control group. The stillbirth rate was 0.51% in the pre-SSc group, 0.57% in the post-SSc group, and 0.08% in the control group. Maternal mortality occurred in 0.45% (n = 8) of the pre-SSc group and 0.08% (n = 2) of the control group (Table 1).

### 3.2. Obstetric and maternal outcomes

#### 3.2.1. Female infertility (N97)

The prevalence of female infertility was significantly higher in women with SSc than in controls in both the pre-SSc (28.04% versus 18.69%; RR 1.70, 95% CI 1.47–1.96) and post-SSc periods (25.25%; RR 1.47, 95% CI 1.21–1.79).

#### 3.2.2. Abortion (O03–O08, N96)

Abortion was more frequent in women with SSc, occurring in 11.70% (n = 207) of the pre-SSc group compared with 7.46% of controls (RR 1.64, 95% CI 1.33–2.03), and in 10.64% (n = 75) of the post-SSc group compared with controls (RR 1.48, 95% CI 1.11–1.96). Multiparity was observed in two patients in the pre-SSc group.

#### 3.2.3. Gestational diabetes (O24)

The prevalence of gestational diabetes was 3.96% (n = 70) in the pre-SSc group and 2.98% (n = 21) in the post-SSc group; the risk was significantly increased in both groups and was more pronounced in the pre-SSc group (RR 6.66 versus 4.96).

#### 3.2.4. Hypertensive disorders of pregnancy (I10–I15, O13–O14)

Hypertensive disorders of pregnancy occurred in 25.55% (n = 452) of the pre-SSc group and 11.06% (n = 78) of the post-SSc group; the risk was significantly increased in both groups and was greater in the pre-SSc group (RR 8.03 versus 2.91).

#### 3.2.5. Puerperal complications (O90)

Puerperal complications were observed in 2.54% (n = 45) of the pre-SSc group and 3.12% (n = 22) of the post-SSc group and were significantly more frequent than in controls in both groups (RR 1.85 and 2.28, respectively). Data on the frequency of pregnancy events observed at least once per patient are presented in Table 2.

#### 3.2.6. Postpartum infections

Breast infections associated with childbirth (O91) were more frequent in both the pre-SSc group (3.05% versus 1.72%; RR 1.80, 95% CI 1.20–2.70) and the post-SSc group (3.83% versus 1.72%; RR 2.27, 95% CI 1.39–3.72) compared with controls. Puerperal infections (O86) were also more frequent in both the pre-SSc group (1.41% versus 0.61%; RR 2.32, 95% CI 1.22–4.41) and the post-SSc group (1.84% versus 0.61%; RR 3.04, 95% CI 1.44–6.41) compared with controls.

### 3.3. Clinical manifestations of systemic sclerosis

Pulmonary hypertension was observed in 1.30% (n = 23) of the pre-SSc group and 2.13% (n = 15) of the post-SSc group, with a higher prevalence in the post-SSc group. Raynaud’s syndrome (I73.0) occurred in 13.11% (n = 232) of the pre-SSc group and 17.59% (n = 124) of the post-SSc group and was more frequent in the post-SSc group. Arthritis was observed in 25.73% (n = 455) of the pre-SSc group and 25.06% (n = 177) of the post-SSc group.

## 4. Discussion

Our results demonstrated an increased risk of pregnancy-related events in patients with SSc compared with the control group. Nevertheless, a considerable proportion of patients were able to achieve successful pregnancy outcomes.

Rheumatic diseases occur frequently in women of reproductive age [11]. Therefore, the consequences of rheumatologic conditions during pregnancy are of particular clinical importance. The management of pregnant patients with SSc may be challenging. The limited availability of data in the literature further highlights the need for evidence to guide clinical practice. To the best of our knowledge, this study includes one of the largest cohorts of pregnancies in patients with SSc.

Vasculopathy is a hallmark feature of SSc. Accordingly, placental vasculopathy may contribute to adverse pregnancy outcomes. Furthermore, major organ-threatening complications such as PAH, interstitial lung disease, and renal crisis may further complicate pregnancy in patients with SSc. In addition, fibrotic processes and tissue dryness may also contribute to complications during labor. These mechanisms remain hypothetical and should be investigated in future mechanistic studies.

Regarding infertility rates, 16.5% of patients in our study had recorded ICD codes for infertility. This rate was higher than those reported by Sampaio-Barros et al. [12] (3.3%) and Steen et al. [13] (5%). Infertility in SSc may occur directly or indirectly due to vasculopathy, disease activity, delayed married, and social stigma related to skin involvement.

Pregnancy loss is an important outcome for both expectant mothers and their families, as well as for healthcare professionals. The frequency of abortion is higher in patients who have experienced at least one pregnancy after the diagnosis of SSc. Ismail et al. reported that approximately 20% of pregnancies do not result in a timely and healthy birth [14]. According to national follow-up data, approximately 13% of all pregnancies result in spontaneous miscarriage. Consistent with previous studies, the abortion rate was significantly higher in our study than in the control group.

The overall maternal age at pregnancy in our study was similar to that reported in Indian and Italian cohorts [15,16]. However, the mean maternal age at pregnancy was lower in the post-SSc group in our study. The rate of cesarean section (CS) delivery was similar between groups in our study. In a study by Eudy et al., the rate of cesarean delivery reached up to 65% in pregnancies with SLE [17]. This may be related to physicians’ concerns regarding difficult labor in patients with SSc; therefore, earlier delivery may have been preferred to ensure favorable outcomes. According to national data from Türkiye, the CS rate in the past 5 years was 57.55% [18]. In this study, CS was more frequent in patients with SSc (69.3%), regardless of the timing of diagnosis.

Preterm labor and birth may occur due to various maternal and fetal complications [19]. Preterm labor may be triggered by multiple mechanisms, including infection, inflammation, uteroplacental ischemia or hemorrhage, and other immunologically mediated processes [20].

Previous studies from different national cohorts have consistently reported increased rates of adverse pregnancy outcomes in patients with SSc [21–23]. However, data on postpartum complications remain limited in the literature. In this context, our findings provide novel evidence that contributes to the existing literature. In our study, postpartum infections, breast-related disorders, and vascular complications were significantly more frequent in the SSc group than in the control group. These results suggest that postpartum morbidity may represent an underrecognized aspect of SSc-related pregnancy outcomes and may be associated with the underlying systemic vasculopathy characteristic of the disease.

A recent metaanalysis on pregnancy in SSc demonstrated higher risks of miscarriage (OR 1.6, 95% CI 1.22–2.22), IUGR (OR 3.2, 95% CI 2.21–4.53), preterm birth (OR 2.4, 95% CI 1.14–4.86), and low birth weight (OR 3.8, 95% CI 2.16–6.56). Patients with SSc had a 2.8-fold higher risk of developing gestational hypertension and a 2.3-fold higher risk of CS compared with controls (OR 2.3, 95% CI 1.37–3.8) [24]. In another study with a design similar to ours, increased relative risks were reported for preeclampsia (RR 3.8, 95% CI 1.8–7.8), preterm birth (RR 3.3; 95% CI 1.8–6.1), and CS (RR 2.5; 95% CI 1.8–3.5) in post-SSc pregnancies. In addition, no increase in maternal or neonatal mortality or stillbirth was reported. Higher risks were reported in primiparous women with post-SSc pregnancies (preeclampsia RR 7.5, 95% CI 3.5–16.1; preterm birth RR 5.1, 95% CI 2.5–10.5) [25]. These findings are consistent with our results.

The limitations of this study include the use of data obtained from the e-Nabız system, which may contain deficiencies due to unregistered or incorrectly coded information; furthermore, ICD coding does not fully reflect the complex nature of diseases, and the retrospective design increases the possibility of bias and error. Disease activity status and the timing of immunosuppressive treatments during pregnancy could not be fully assessed in this study. Furthermore, autoantibody profiles, SSc subtypes (diffuse and limited), treatment histories, and involved organ systems could not be clearly obtained and were therefore not included. Another limitation is that the true onset of disease may not have been captured, as data were only available from a specific time point onward. Lastly, multivariate analyses to evaluate the effects of other parameters, such as smoking and comorbidities, could not be performed due to limited data availability and uncertainty regarding the timing of comorbidity onset. These factors may have significant impacts on the generalizability and interpretation of the study results. Nevertheless, nationwide coverage, a large sample size, a matched control group, and the comparison of prediagnosis and postdiagnosis pregnancies represent major strengths of this study.

In conclusion, this study represents one of the largest and most comprehensive retrospective analyses of pregnancy outcomes in patients with SSc. Despite these limitations, our findings provide important insights into pregnancy outcomes in SSc. It should be acknowledged that certain risks for adverse outcomes are increased in SSc pregnancies; however, with appropriate preconception planning and multidisciplinary high-risk obstetric care, successful pregnancy and delivery are possible for many women with SSc. Case-by-case evaluation and prospective studies are needed to provide more precise evidence to guide clinical practice.

## Figures and Tables

**Table 1:** Number of patients who experienced at least one pregnancy-related complication in both groups.

	Pre-SSc		Control			Post-SSc		Control		
	N	%	N	%	RR (%95 CI)	N	%	N	%	RR (%95 CI)
Infertility	496	28.04	456	18.69	1.7 (1.47–1.96)	178	25.25	456	18.69	1.47 (1.21–1.79)
Ectopic pregnancy	119	6.73	108	4.43	1.56 (1.19–2.04)	53	7.52	108	4.43	1.76 (1.25–2.47)
Bleeding during pregnancy	482	27.25	444	18.20	1.68 (1.45–1.95)	197	27.94	444	18.20	NA
Abortion	207	11.70	182	7.46	1.64 (1.33–2.03)	75	10.64	182	7.46	1.48 (1.11–1.96)
Hydatidiform mole	1	0.06	4	0.16	0.34 (0.04–3.08)	1	0.14	4	0.16	0.87 (0.1–7.75)
Placenta previa	8	0.45	7	0.29	1.58 (0.57–4.36)	3	0.43	7	0.29	1.49 (0.38–5.76)
Placental disorders	2	0.11	1	0.04	2.76 (0.25–30.47)	0	0.00	1	0.04	NA
Multiple pregnancy/delivery	2	0.11	14	0.57	0.2 (0.05–0.86)	0	0.00	14	0.57	NA
Venous complications during pregnancy	57	3.22	48	1.97	1.66 (1.13–2.45)	16	2.27	48	1.97	1.16 (0.65–2.05)
Gestational diabetes mellitus	70	3.96	15	0.61	6.66 (3.8–11.67)	21	2.98	15	0.61	4.96 (2.55–9.68)
Hypertensive disorders of pregnancy	452	25.55	100	4.10	8.03 (6.4–10.08)	78	11.06	100	4.10	2.91 (2.14–3.97)
Edema and proteinuria in pregnancy and the puerperium	26	1.47	4	0.16	9.08 (3.17–26.08)	15	2.13	4	0.16	13.24 (4.38–40.02)
Eclampsia	1	0.06	0	0.00	NA	0	0.00	0	0.00	NA
Puerperium-related complications	141	7.97	110	4.51	1.84 (1.42–2.37)	71	10.07	110	4.51	2.37 (1.74–3.24)
Puerperal infections	25	1.41	15	0.61	2.32 (1.22–4.41)	13	1.84	15	0.61	3.04 (1.44–6.41)
Venous complications in the puerperium	10	0.57	4	0.16	3.46 (1.08–11.06)	7	0.99	4	0.16	6.11 (1.78–20.92)
Puerperium-related complications	45	2.54	34	1.39	1.85 (1.18–2.9)	22	3.12	34	1.39	2.28 (1.32–3.92)
Breast infections associated with childbirth	54	3.05	42	1.72	1.8 (1.2–2.7)	27	3.83	42	1.72	2.27 (1.39–3.72)
Disorders of the breast and lactation associated with childbirth	10	0.57	15	0.61	0.92 (0.41–2.05)	12	1.70	15	0.61	2.8 (1.3–6.01)

N/A: insufficient data for relative risk calculation.

**Table 2:** Birth outcomes and Robson classification values.

	Pre-SSc (n = 1769)		Post-SSc (n = 705)		Control (n = 2440)		Total (n = 4914)	
	N	%	N	%	N	%	N	%
**Age**								
Maternal age, median (IQR)	30 (8)		30 (7)		30 (8)		30 (8)	
Diagnosis age, median (IQR)	26 (8)		33 (8)			28 (9)		
**Birth weight (g)**								
≤2500 g	208	11.76	71	10.07	119	4.88	398	8.10
≥2500 g	1561	88.24	634	89.93	2321	95.12	4516	91.90
**Robson group**								
Group 1	510	28.83	168	23.83	529	21.68	1207	24.56
Group 2	79	4.47	25	3.55	80	3.28	184	3.74
Group 3	335	18.94	151	21.42	579	23.73	1065	21.67
Group 4	42	2.37	21	2.98	94	3.85	157	3.19
Group 5	413	23.35	217	30.78	763	31.27	1393	28.35
Group 6	64	3.62	16	2.27	80	3.28	160	3.26
Group 7	33	1.87	19	2.70	93	3.81	145	2.95
Group 8	32	1.81	4	0.57	29	1.19	65	1.32
Group 9	34	1.92	15	2.13	49	2.01	98	1.99
Group 10	227	12.83	69	9.79	144	5.90	440	8.95
**Gestational age at birth**								
Preterm	279	15.77	84	11.91	178	7.30	541	11.01
Term	1483	83.83	621	88.09	2256	92.46	4360	88.73
Postterm	7	0.40	0	0.00	6	0.25	13	0.26
**Mode of delivery**								
Vaginal birth	570	32.22	235	33.33	792	32.46	1597	32.50
Cesarean section	1199	67.78	470	66.67	1648	67.54	3317	67.50
**Birth outcome**								
Live birth	1760	99.49	701	99.43	2438	99.92	4899	99.69
Stillbirth	9	0.51	4	0.5	2	0.08	15	0.31
**Maternal mortality**								
No	1761	99.55	705	100	2438	99.92	4899	99.69
Yes	8	0.45	-	-	2	0.08	10	0.31

## Data Availability

All data are held by the General Directorate of Information Technologies of the Ministry of Health of the Republic of Türkiye.
